# 伴再生障碍性贫血表现T大颗粒淋巴细胞白血病5例报告并文献复习

**DOI:** 10.3760/cma.j.issn.0253-2727.2023.02.015

**Published:** 2023-02

**Authors:** 小霞 李, 建平 李, 馨 赵, 园 李, 佑祯 熊, 广新 彭, 蕾 叶, 文睿 杨, 康 周, 慧慧 樊, 洋 杨, 洋 李, 琳 宋, 丽萍 井, 莉 张, 凤奎 张

**Affiliations:** 中国医学科学院血液病医院（中国医学科学院血液学研究所），实验血液学国家重点实验室，国家血液系统疾病临床医学研究中心，细胞生态海河实验室，天津 300020 State Key Laboratory of Experimental Hematology, National Clinical Research Center for Blood Diseases, Haihe Laboratory of Cell Ecosystem, Institute of Hematology & Blood Diseases Hospital, Chinese Academy of Medical Sciences & Peking Union Medical College, Tianjin 300020, China

T大颗粒淋巴细胞白血病（T-LGLL）是一类少见的以伴发自身免疫性疾病及骨髓髓系造血损伤为主要特点的淋巴细胞增殖性疾病，多数患者呈惰性临床病程，无任何临床症状，部分患者表现一系或多系的血细胞减少、感染及自身免疫性疾病等。T-LGLL表现为纯红细胞再生障碍较为常见，而表现为AA罕有报道。我们回顾性分析我院近5年收治的5例表现为AA的T-LGLL患者的临床特征及治疗转归，现报道如下。

## 病例与方法

回顾性分析2015年2月至2020年11月我院门诊及贫血诊疗中心明确诊断T-LGLL且临床表现为AA的患者临床及实验室检查资料，包括临床表现、血常规、免疫表型、形态学检查、分子生物学检查、治疗方案及治疗反应等。T-LGLL的诊断标准[1]–[3]：①外周血T细胞大颗粒淋巴细胞（T-LGL）增多，绝对值>0.5×10^9^/L；②外周血或骨髓涂片可见到较多的LGL，流式细胞术检测外周血或骨髓中异常增殖的细胞群免疫表型符合T-LGL；③PCR检测到TCRβ/γ基因重排阳性或流式细胞术检测到TCRVβ亚家族呈单克隆增殖；④临床表现包括血细胞减少、脾大、类风湿关节炎以及B症状（发热、盗汗、体重减轻）等。诊断T-LGLL需满足以上4条标准。若符合②③④条标准，但骨髓病理可见到T-LGL浸润（骨髓间质或窦状隙内簇状出现至少8个CD8^+^或TIA-1^+^ LGL，或至少6个颗粒酶B^+^ LGL）也可明确T-LGLL的诊断。AA的诊断及分型参照文献[4]标准，T-LGLL与AA确诊时间间隔<3个月定义为同时发病，确诊时间间隔≥3个月定义为先后发病。治疗后6、12个月进行疗效评价，根据AA的疗效标准[4]判定。

## 结果

1. 患者一般特征：见表1。5例患者中男3例，女2例，中位年龄40（27～49）岁。其中2例为输血依赖的非重型再生障碍性贫血（TD-NSAA），2例为重型再生障碍性贫血（SAA），1例为极重型再生障碍性贫血（VSAA）。例1确诊T-LGLL 7年后出现AA样表现，其余4例起病时即呈现AA的临床特征。骨髓象均显示：增生减低，粒红系明显减少，巨核细胞少见或缺如，LGL形态的淋巴细胞增多，无明显病态造血形态学改变；骨髓活检均显示增生减低。5例患者均为正常染色体核型。4例患者完善了骨髓细胞二代测序，其中2例检出STAT3突变（c.1919A>T/p.Y640F）。LGL免疫表型2例为CD4^+^CD8^dim^，3例为CD4^−^CD8^+^；4例为αβ型T细胞，1例为γδ型T细胞。所有患者均无PNH克隆。5例患者中有2例抗核抗体阳性，而类风湿因子均阴性。所有患者均无淋巴结或肝脾增大；均未合并其他血液及实体肿瘤。

**表1:** 5例伴再生障碍性贫血（AA）表现T-LGLL患者的临床特征、治疗及转归

例号	年龄（岁）	性别	就诊原因	AA表现	WBC（×10^9^/L）	ANC（×10^9^/L）	HGB（g/L）	ALC（×10^9^/L）	LY（％）	PLT（×10^9^/L）	ARC（×10^9^/L）	LGL计数（×10^9^/L）
1	40	女	T-LGLL诊治病史7年，全血少2个月	SAA	2.14	0.72	91	1.25	58	12	19.0	0.44
2	27	男	皮肤出血点3个月	VSAA	3.70	0.16	60	3.52	95	15	5.4	0.97
3	45	男	皮肤出血点3个月	SAA	3.37	2.37	78	1.14	34	11	18.2	0.53
4	29	女	全血少1年余	TD-NSAA	2.30	0.65	63	1.49	65	9	43.4	0.48
5	49	男	血小板减少2年余	TD-NSAA	2.85	1.21	90	1.43	50	3	29.0	0.52

例号	骨髓增生程度	免疫表型	TCRVβ（％）	TCR重排（β/γ）	STAT3突变频率（％）	治疗方案	6个月疗效
1	<10%	CD3^+^CD4^−^CD5^dim^CD7^dim^CD8^dim^、CD16^+^CD56^−^CD57^p+^TCRαβ^+^	58.8	β^+^γ^−^	10.9	HD-CTX	PR
2	<10%	CD3^+^CD4^−^CD5^−^CD7^dim^、CD8^+^CD56^dim^CD57^+^TCRγδ^+^	-	β^−^γ^+^	野生型	ATG+CsA	CR
3	<10%	CD2^+^CD3^+^CD4^+^CD5^++^CD7^dim^CD8^dim^CD57^+^TCRαβ^+^	89.8	β^+^γ^+^	NA	ALG+CsA	CR
4	30%	CD3^+^CD4^+^CD5^+^CD7^dim^CD8^dim^CD16^−^CD56p^+^CD57^+^TCRαβ^+^	88.1	β^+^γ^+^	野生型	CsA单药，后转为泼尼松+沙利度胺	NR
5	<10%	CD3^+^CD4^−^CD5^dim^CD7^−^CD8^dim^CD16^−^CD56^−^CD57^+^TCRαβ^+^	97.4	β^+^γ^+^	4.5	CsA+艾曲泊帕+泼尼松	PR

**注** T-LGLL：T大颗粒淋巴细胞白血病；SAA：重型再生障碍性贫血；VSAA：极重型再生障碍性贫血；TD-NSAA：输血依赖非重型再生障碍性贫血；ALC：淋巴细胞绝对计数；LY：淋巴细胞比值；ARC：网织红细胞绝对计数；LGL：大颗粒淋巴细胞；HD-CTX：大剂量环磷酰胺；ATG：抗胸腺细胞免疫球蛋白；CsA：环孢素A；PR：部分治疗反应；CR：完全治疗反应；NR：无治疗反应；-示TCRVβ阴性；NA示未检测

2. 疗效与转归：5例患者中1例（例1）接受大剂量环磷酰胺（HD-CTX）30 mg·kg^−1^·d^−1^×4 d治疗，4例接受免疫抑制治疗，具体用药方案见表1，6个月时4例有效［完全治疗反应（CR）2例、部分治疗反应（PR）2例］。例1在治疗后12个月时疗效评估为CR；例4应用环孢素A（CsA）单药治疗4个月后疗效评估为无治疗反应（NR），后调整为糖皮质激素联合沙利度胺，治疗2个月后评估疗效仍为NR；例5应用CsA联合泼尼松及艾曲泊帕（艾曲泊帕应用4个月后停用）疗效评估为PR。中位随访20（7～521）个月，5例患者仍有克隆性T-LGL存在，但血象均稳定。

## 讨论

T-LGLL是一种慢性淋巴细胞增殖性疾病，多见于老年人，临床病程隐匿，多数患者无明显症状和体征，常于体检时发现淋巴细胞增多，部分患者因贫血或感染相关症状而就诊，血小板计数往往正常或仅有轻度减少，骨髓活检可以是增生增高、正常或低下，外周血或骨髓可检测到克隆性LGL增多[5]。本文报道的5例患者，青壮年起病，均以出血为主要症状，外周血表现全血细胞减少，骨髓增生减低，无病态造血，外周血和或骨髓可检测到克隆性LGL增多，呈现AA的临床特征，并符合T-LGLL的诊断标准。在获得性AA患者中也可出现T淋巴细胞克隆性增生[6]–[7]，但仅有TCR重排阳性并不足以确诊T-LGLL，尚需要满足外周血LGL数量的要求或具有LGL侵犯骨髓的病理学证据。

目前认为AA是由于细胞毒性T淋巴细胞（CTL）异常活化、功能亢进生成大量细胞因子抑制造血，并引起造血干细胞表面Fas受体表达增加，CTL表面Fas L与Fas受体相互作用导致了靶目标造血干细胞过度凋亡，然而这种异常免疫究竟是由于造血干细胞本身异常驱动，反过来引起造血衰竭，还是由于其他抗原触发导致骨髓衰竭，目前尚未明确[8]–[9]。T-LGLL的发病现在认为是由慢性抗原刺激，激活存活信号通路和抑制凋亡信号通路，使得“LGL样”的CTL持续增殖及活化。T-LGLL与获得性AA具有共同的免疫基因背景，包括STAT3和HLA-DR15易感[10]，均存在T淋巴细胞的异常活化，且免疫抑制治疗于两者均有效，推测两种疾病可能存在相似的发病机制。本文中例1继T-LGLL确诊7年后出现AA样的临床特征，提示可能是由于克隆性T-LGL异常免疫进展而诱发了造血功能衰竭，也有可能是免疫抑制治疗T-LGLL引起的“免疫失调节作用”诱发AA样表现。但从本文另外4例及文献报道的病例[7]来看，似乎更多的患者表现T-LGLL与AA样表现同时发生，提示AA样表现可能是T-LGLL的一种特殊临床表现型。然而以上假设是否成立尚需研究证实。

免疫表型检测是鉴定T-LGL的关键之一，T-LGLL根据TCR类型不同可分为αβT-LGLL和γδT-LGLL，临床上以αβT-LGLL多见，约占CD3^+^ T-LGLL的95％，免疫表型多为CD3^+^CD4^−^CD8^+^CD57^+^TCRαβ^+^，称之为经典型T-LGL免疫表型；γδT-LGLL较为少见，以CD4^−^CD8^−^双阴性免疫表型更为多见[11]。我中心曾总结过108例T-LGLL患者的临床特点，16％患者为γδT-LGL表型，CD4^−^CD8^−^及CD4^+^CD8^−^的免疫表型更为少见，分别为5例和1例[12]。我们回顾最近5年我院门诊及住院治疗的T-LGLL中仅发现5例表现AA，其中有3例患者为少见表型（1例为CD4^−^CD8^+^TCRγδ，2例为CD4^+^CD8^−^TCRαβ），少见表型CD4^+^CD8^−^ T细胞克隆性扩增表现AA的机会更多。目前关于T-LGLL表现为AA的病例以个案报道多见，偶见小样本回顾分析，T-LGL的免疫表型与AA是否存在相关性尚未有文献报道。Go等[7]曾报道9例表现为AA的T-LGLL病例，是目前为止报道例数最多的，但因条件限制未进一步标记LGL的免疫表型，无法获得患者免疫表型数据。王兰等[13]报道一例少见表型CD4^−^CD8^−^CD56^+^γδT-LGLL表现为AA的案例。另外，文献报道T-LGLL也可呈现骨髓增生异常综合征（MDS），但较少对其免疫表型进行描述，我院石茵等[14]报道3例MDS合并克隆性T-LGL增殖的病例，T-LGL均为少见表型，1例为CD4^−^CD8^+^TCRγδ，另2例为CD4^+^CD8^−^TCRαβ，表型与本研究相一致。目前认为CD4^+^ T-LGLL患者多无临床症状，但常合并其他肿瘤[15]–[18]。但由于样本量的限制，是否少见免疫表型的T-LGLL有其特殊的临床特点尚需要临床经验的积累及总结。

异常的STAT3信号是维持LGL克隆扩增的关键，T-LGLL常伴有STAT3基因突变，突变率为28％～40％，STAT3突变型患者血细胞计数（ANC、RBC）低于野生型患者，突变型患者常需要接受治疗，且对于免疫抑制治疗反应佳[19]–[20]。有报道发现获得性AA患者也可出现STAT3基因突变，突变率约为11％（16/140），且STAT3突变发生于Vβ^+^ T细胞，因此提出了STAT3突变的T-LGL克隆诱发AA的假说。研究还发现伴STAT3突变的AA患者对于一线免疫抑制治疗的反应似乎更好[10]。本组T-LGLL患者中有一半（2/4）伴有STAT3突变，STAT3突变型的患者对于免疫治疗均有效，但由于样本量少，尚需要扩大样本量进一步明确STAT3突变型T-LGLL伴AA表现患者的临床及治疗疗效特点。

IST是目前公认的对于不适宜行造血干细胞移植的SAA患者的一线治疗方案，可使60％～80％的SAA患者造血恢复[21]。另外，我中心应用HD-CTX治疗复发或CsA耐药的T-LGLL及初治/难治/复发V/SAA，并获得较为满意的疗效[12],[22]–[23]，本组患者中亦显示IST方案疗效最佳，HD-CTX也获得了极好的疗效。鉴于AA样表现的T-LGLL与获得性AA有相似的发病机制、临床表现及治疗疗效，其治疗方案可参照获得性AA。

综上，AA样表现更多见于非典型免疫表型的T-LGLL，此类患者的治疗应参照获得性AA。伴AA表现的T-LGLL的案例提示LGL克隆扩增可能是触发骨髓造血功能衰竭的原因之一，其可能的内在机制值得进一步的研究探讨。
